# Dynamin-related protein 1 controls the migration and neuronal differentiation of subventricular zone-derived neural progenitor cells

**DOI:** 10.1038/srep15962

**Published:** 2015-10-30

**Authors:** Hyun Jung Kim, Mohammed R. Shaker, Bongki Cho, Hyo Min Cho, Hyun Kim, Joo Yeon Kim, Woong Sun

**Affiliations:** 1 Department of Anatomy, Brain Korea 21 Plus Program, Korea University College of Medicine, Seoul, 136-705, Republic of Korea; 2 Department of Brain Science, Daegu Gyeongbuk Institute of Science and Technology, Daegu, Republic of Korea

## Abstract

Mitochondria are important in many essential cellular functions, including energy production, calcium homeostasis, and apoptosis. The organelles are scattered throughout the cytoplasm, but their distribution can be altered in response to local energy demands, such as cell division and neuronal maturation. Mitochondrial distribution is closely associated with mitochondrial fission, and blocking the fission-promoting protein dynamin-related protein 1 (Drp1) activity often results in mitochondrial elongation and clustering. In this study, we observed that mitochondria were preferentially localized at the leading process of migratory adult neural stem cells (aNSCs), whereas neuronal differentiating cells transiently exhibited perinuclear condensation of mitochondria. Inhibiting Drp1 activity altered the typical migratory cell morphology into round shapes while the polarized mitochondrial distribution was maintained. With these changes, aNSCs failed to migrate, and neuronal differentiation was prevented. Because Drp1 blocking also impaired the mitochondrial membrane potential, we tested whether supplementing with L-carnitine, a compound that restores mitochondrial membrane potential and ATP synthesis, could revert the defects induced by Drp1 inhibition. Interestingly, L-carnitine fully restored the aNSC defects, including cell shrinkage, migration, and impaired neuronal differentiation. These results suggest that Drp1 is required for functionally active mitochondria, and supplementing with ATP can restore the defects induced by Drp1 suppression.

Mitochondria mediate many essential cellular functions required for the survival and maintenance of cell, as mitochondria play multiple roles in the energy production and calcium homeostasis. Accordingly, impaired mitochondria are frequently found in diseases, which are associated with organs that require high energy consumption, such as the brain^1^. In many cell types, mitochondria are spread throughout the cytoplasm, and their distribution is under tight control depending on the cellular processes, such as cell division, cell attachment, and migration. In developing neurons, mitochondria also accumulate in subcellular regions that require high energy demands, such as active growth cones^2^,^3^. Following neuron maturation, mitochondria are also localized at presynaptic terminals where they generate ATP and modulate synaptic calcium concentration, which are essential for proper synaptic functions and plasticity^4^,^5^,^6^,^7^. Therefore, the localization of mitochondria is dynamically regulated by the process of neuronal maturation.

Mitochondria are highly mobile and dynamic organelles that move along the cytoskeletal tracks via motor proteins^8^. Because mitochondria form highly tubular/network shapes in many quiescent cells, mitochondria fragmentation is required for efficient subcellular redistribution^9^. Dynamic changes of mitochondrial morphology are governed by mitochondrial fission and fusion. Mitochondrial fission is mainly executed by the GTPase-dependent squeezing of dynamin-related protein 1 (Drp1) on the cytosolic surface of the mitochondrial outer membrane, whereas fusion is mediated mainly by two different molecules, Mfn1/2 and Opa1. Blocking Drp1 often prevents mitochondrial dispersion, causing mitochondria to cluster in perinuclear regions^10^. Hypoxic conditions, for example, also promote perinuclear clustering of mitochondria through inactivated Drp1, and the local production of reactive oxygen species (ROS) appears to mediate hypoxia-dependent cellular events, suggesting that mitochondrial fission machinery is involved in the local energy and/or ROS production in the cell^11^.

Changes in Drp1 expression directly influence cellular metabolism and ultimately cell fate. During myogenic differentiation, increased Drp1 expression and its translocation to mitochondria are required for the metabolic shift from glycolysis to oxidative phosphorylation^12^,^13^. Similarly, Drp1 is required for neuronal differentiation. In embryonic stem cells, knockdown of Drp1 reduces embryoid body formation and differentiation into neural lineages^14^. In brain tumor initiating cells, Drp1 hyperactivation results in maintaining self-renewal and tumor formation, and decreasing activation of Drp1 phosphorylation (Ser616) is observed during differentiation^15^. The developmental potential of maturing mouse oocyte is also related to redistributing mitochondria via Drp1 from the cell periphery to perinuclear clustering^16^, suggesting that Drp1 activity controls the mitochondrial distribution that is required for cellular differentiation/maturation.

Migration, as well as differentiation, is critical for many biological and pathological events, such as morphogenesis during embryonic development, migration of immune cells, wound healing, and invasion of cancer cells. Cell migration requires a polarized morphology with an asymmetrical distribution of cytoskeletal components and organelles. For example, mitochondria accumulate at the rear end of lymphocytes to provide ATP during chemotaxis^17^. In cancer cells, relocation of mitochondria also plays a role in migration and invasion^18^,^19^. Perturbations of mitochondrial fission and fusion induced a decrease in speed and persistence of migration, suggesting that regulating mitochondrial dynamics influences the transportation and relocation of mitochondria, and thereby, the ability of cells to move forward.

Since proper migration and differentiation are fundamental for aNSCs to construct the brain structure during development, thus, we hypothesized that mitochondrial dynamics and localization are necessary to maintain aNSC migration and differentiation. To this end, we explored the mitochondrial distribution in aNSCs, and the impact of Drp1 suppression on aNSC migration and differentiation.

## Materials and Methods

### Neurosphere culture

Neurosphere cultures were established from the anterior subventricular zone (SVZ) of 8- to 9-week-old male C57BL/6 mice (Orient Bio Inc., Seongnam, Korea) as described before by Moon *et al*.^20^. Brains were removed and cut into thick coronal brain slices using a brain matrix. Freshly isolated SVZ tissues were first digested with 0.8% papain (Worthington, Lakewood, NJ, USA) and 0.08% dispase II (Roche Applied Science, Indianapolis, IN, USA) in Hank’s Balanced Salt Solution (HBSS), followed by 45 min of incubation in 37 °C water bath. Dissociated cells were plated onto an ultra-low attachment six-well plate for generating neurospheres in DMEM/F12 media supplemented with 1% N2, 2% B27 and 1% antibiotic-antimycotic (Invitrogen, Carlsbad, CA, USA). Neurospheres were maintained by the daily addition of 20 ng/ml epidermal growth factor (EGF) and basic fibroblast growth factor (bFGF). At days *in vitro* (DIV) 8, primary neurospheres were dissociated with Accutase (Innovative Cell Technologies, San Diego, CA, USA) and subsequently subcultured every 4–5 days up to 5 passages. For the migration assay, similar sizes of neurospheres were plated onto poly-D-lysine and laminin coated cover slip in complete media containing EGF/bFGF. For differentiation, attached neurospheres were treated with Mdivi-1 and/or L-carnitine 3 hours after plating and allowed to differentiate for 6 days in the media without EGF/bFGF. We analyzed the differentiated cells at the margin of neurospheres from 3 independent experiments. The number of neurospheres examined was indicated in each figure. All experiments were carried out in accordance with the ethical guidelines of Korea University and with the approval of the Animal Care and Use committee of Korea University.

### Immunofluorescence, image acquisition and analysis

Cells were loaded with 25 nM MitoTracker Red (Molecular Probes) for 15 min to stain mitochondria. Alternatively, Immunostaining was performed according to Shaker *et al*.^21^, cells were fixed with 4% paraformaldehyde, blocked with 3% Bovin Serum Albumin (BSA) and 0.1% Triton X-100 in phosphate buffered saline (PBS) for 1 hour, and incubated with the following primary antibodies at 4 °C overnight: anti-Cytochrome C (1:500; BD Bioscience), anti-Drp1 (1;500, BD Bioscience), anti-Nestin (1:1000; Millipore), anti-DCX (1:500; Santa Cruz), anti-GTU88 (1:500; Sigma Aldrich), anti- beta-Tubulin III (1:1000; Sigma Aldrich), anti-glial fibrillary acid protein (GFAP) (1:500; Invitrogen). Samples were incubated with subsequent secondary antibodies at room temperature for 1 hour. To label F-actin, cells were fixed and stained with Alexa Fluor 488-labeled Phalloidin, and the nuclei were counter-stained with Hoechst 33342. Images were taken with a confocal microscope (Zeiss LSM700) or EVOS FL Cell Imaging System (Life Technologies).

For polarity, mitochondria at the leading edge were assessed by measuring the distribution of cell area away from the nuclei, as described by Pryor *et al*.^22^. Eight circular sectors were applied to the center of the nucleus to measure the mitochondrial distribution at the front, back, left, and right sides of each cell. Polarity analysis was performed on 30 to 40 cells from at least three independent experiments. All area and distance measurements were performed using ImageJ software (NIH).

### Neurosphere migration and video analysis

Neurospheres with similar sizes (Ranged 180–210 μm) were seeded on plates coated with poly-D-lysine and laminin in medium containing EGF/bFGF with or without the Drp1 selective inhibitor mitochondrial division inhibitor (Mdivi-1) and/or L-carnitine for the migration assay.

The neurospheres attached and began to migrate approximately 2 hours after plating, and the Mdivi-1 and/or L-carnitine were treated at the concentration of 50 nM as indicated in the results. Images were taken after 24 hours of neurosphere seeding by EVOS FL Cell Imaging System (Life Technologies). Lines were drawn onto the longest axis and its perpendicular axis of the neurosphere, and the migration lengths were measured from the axis crossing the core of the neurosphere using Image J. The maximum migration lengths were used to form a graph. To measure outgrowth, the rate of cell migration over 24 hours was assessed by measuring the area of outgrowth as a percentage of increase in the area covered by the central neurospheres core at 2 hours. To monitor migration in real-time, neurospheres were seeded onto the coated plate as described above and treated with Mdivi-1 at 2 hours. At 6 hours after plating, migrating cells were monitored for 1 hour using JuLi (NanoEntek, Seoul, Korea). Resulting time-lapse images were analyzed to assess the directionality and velocity using Chemotaxis and Migration Tool Version 1.01 (Ibidi).

### Transfection

DsRed-mito and pIRES2-GFP clones were purchased from Clontech. DN-DRP1 (K38A) IRES-GFP was generated based on a previous experiment^23^, with the exception of using dsRed-mito in place of GFP in the DN-DRP1 (K38A) clone to generate DN-DRP1 (K38A) IRES-dsRed-mito. aNSCs (adult NSCs) were divided into two groups: a control group co-electroporated with dsRed- mito and pIRES2-GFP clones, and a DN-DRP1 group co-electroporated with DN-DRP1 (K38A) IRES-dsRed-mito and pIRES2-GFP. For electroporation, 3 × 10^6^ aNSCs were suspended in buffer solution (Nucleofactor Kit, Lonza) with 2–5 μg/μl of DNA constructs. Cubic tubes were then placed in an Amaxa machine and electroporation determined using a program designed for mouse aNSCs. The aNSCs were allowed to expand into neurospheres before being seeded onto coverslips coated with poly-D-lysine (PDL) and laminin.

### Measurement of mitochondrial membrane potential

Mitochondrial membrane potential was measured by tetramethylrhodamine, methyl ester (TMRM) staining (Invitrogen). Cells were incubated in Tyrode’s buffer (145 mM NaCl, 5 mM KCl, 10 mM glucose, 1.5 mM CaCl2, 1 mM MgCl2, and 10 mM HEPES; pH 7.4) containing 10 nM TMRM for 45 min. Images were taken prior to the treatment for baseline fluorescence intensity to detect any changes in TMRM intensity after Mdivi-1 treatment. Additional images were taken after injecting Mdivi-1 at a final concentration of 50 μM. Live imaging of cells was performed by inverted fluorescence microscope (Carl Zeiss Observer Z1), and cells were maintained at 37 °C with 5% CO_2_. Time-lapse images were obtained for 30 min at an interval of 30 sec by MetaMorph software (Molecular Devices).

### Measurement of mitochondrial respiration

Oxygen consumption rate (OCR) and extracellular acidification rate (ECAR) were measured using an XF24 extracellular analyzer (Seahorse Bioscience, Villerica, MA, USA). SVZ-derived aNSCs were seeded in XF24-well microplates at 1x10^5^ cells per well and incubated at 37 °C with 5% CO_2_ overnight. Cells were pre-incubated with Mdivi-1 for 3 hours, and controls were treated with the same amount of Dimethyl sulfoxide (DMSO). Cells were then treated with XF assay media and incubated at 37 °C for 1 hour without CO_2_. Three baseline measurements were taken before the injection of oligomycin (1 μM), and three readings were taken after adding oligomycin. The OCR and ECAR were automatically calculated using the Seahorse software. ATP production of OCR and oligomycin sensitive were measured by calculating the differences of levels between basal and after oligomycin treatment, as described before^24^. Each point represents an average of three different wells of each independent experiment, at least 3 independent experiments were examined.

### Statistical analysis

Data represent the mean ± standard deviation (s.d.) of the mean (SDM). Total number of analyzed neurospheres are indicated in bar graphs of different experiments. Statistical analysis was carried out using Sigma Plot 12. Statistical comparisons were made using Student’s t-test and one-way ANOVA. *P* < 0.05 was considered to be significant.

## Results

### Mitochondrial localization in the leading process of migrating aNSCs

**Figure 1:**
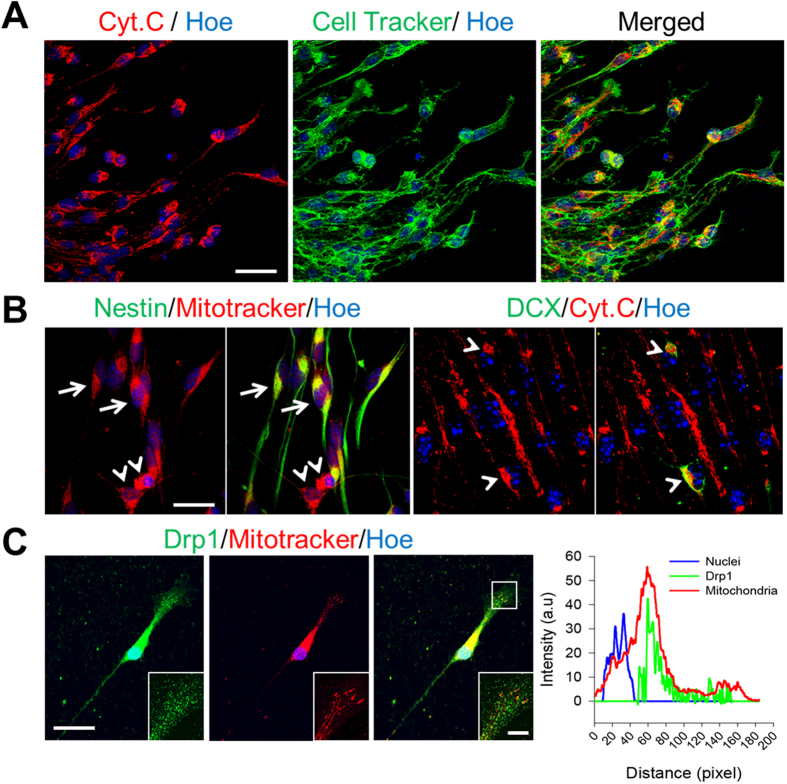
Distribution of mitochondria in cells derived from SVZ neurospheres. (**A**) The morphology of cells and mitochondria were doubly-labeled by cell tracker (green) and cytochrome C (red). Scale bar = 30 μm. (**B**) Mitochondrial distributions in Nestin^+^ (green, arrows) aNSCs labeled by Mitotracker (red, left), and in DCX^+^ (green, arrowheads) neuroblasts labeled by cytochrome C (red, right). Nuclei were counter-stained with Hoechst33342 (blue). Scale bar = 20 μm. (**C**) Distribution of mitochondrial fission protein Drp1 within a cell. Scale bar = 20 μm. Insets show a magnified view of the boxed area. Scale bar in inset = 5 μm. The intensity graph shows that Drp1 co-localized with mitochondria at the leading process. Nuclei were counter-stained with Hoechst33342 (blue).

During neuronal progenitor migration, cells elongate and extend lamellipodia at the leading/front edge. Although a monolayer culture of neural stem/progenitor cells does not promote directional migration, attaching neurospheres onto coverslips triggers the radial migration of cells. In this condition, most cells localized at the periphery of neurospheres exhibited an elongated morphology with lamellipodia formation at the front edge (Fig. 1A). Mitochondria exhibited a polarized distribution at the leading process along the migratory axis. In addition to these migrating cell populations, there were additional cells which showed multipolar morphology and mitochondria predominantly localized in the perinuclear areas. Double staining of Nestin or DCX revealed that mitochondria were polarized within Nestin^+^ aNSCs, whereas clumped mitochondria were seen in DCX^+^ neuronal progenitors (Fig. 1B). We visualized mitochondria with cytomchrome C or Mitotracker which gave rise to the similar quality of mitochondria morphology (data not shown). To further examine mitochondrial distribution and the migratory behaviors *in vitro*, we performed a live imaging experiment, where dsRed-mito-electroporated aNSCs were left to migrate for 2 hours (Supplementary Figure 1). Live imaging data indicated that aNSC with elongated morphology and dispersed mitochondria exhibited migratory potential (Supplementary Figure 1, white arrows), whereas cells with condensed/clumped mitochondria did not move/migrate during the observational periods (Supplementary Figure 1, white arrows head). These results suggest that mitochondria relocation is associated with aNSC differentiation and migratory potential.

Previous reports revealed that Drp1 has a primarily large cytosolic distribution and small membrane-bound fraction in healthy mammalian cells^10^,^25^,^26^. To localize endogenous Drp1 in migrating aNSCs, we double-labeled the cells with Drp1 antibody and Mitotracker. We found that Drp1 was diffused within the cytosol and the punctate form of Drp1 co-localized with mitochondria at the leading processes (Fig. 1C). Thus, Drp1 was distributed preferentially in the anterior region of cells following mitochondrial localization along the migratory axis.

### Suppression of mitochondrial fission alters mitochondrial localization and cell polarity

**Figure 2:**
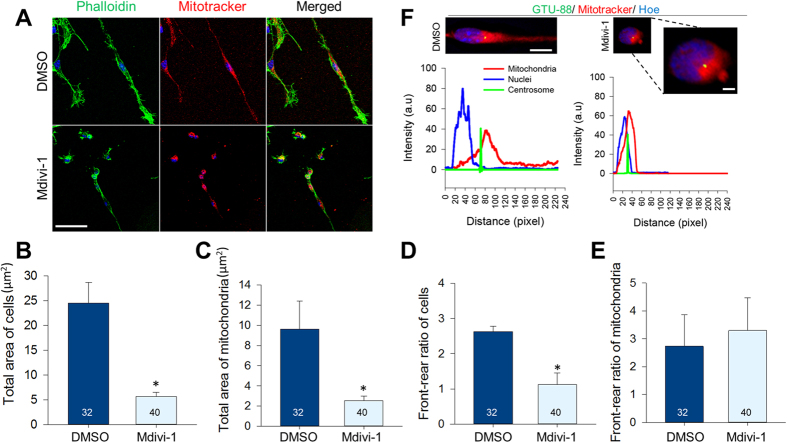
Effects of inhibiting mitochondrial fission on mitochondrial localization and cell polarity. (**A**) Migrating cells form attached neurospheres after treating with DMSO and Mdivi-1. Phalloidin (green) was used to mark cell morphology and Mitotracker (red) to trace mitochondrial distribution. Scale bar = 20 μm. Measurements of the (**B**) total area of cells, (**C**) total area of mitochondrial, (**D**) front-to-rear ratio of cells, (**E**) and mitochondria following Mdivi-1 treatment. Data are shown as mean ± s.d; *P < 0.05 via Student’s t-test. (**F**) Migrating aNSCs doubly-stained with Mitotracker and centrosome (GTU-88, green) to mark the leading edge. Scale bar = 15 μm. The intensity graph shows the distribution of nuclei (blue), mitochondria (red), and centrosome (green). Dotted lines indicate magnified images of Mdivi-1 group. Scale bar = 10 μm. Numbers in bars indicate total number of analyzed cells from 3 independent experiments.

To test whether Drp1 is required to form cellular polarity in migrating aNSCs, we treated neurospheres with 50 μM of Mdivi-1 and found that the migrating aNSCs from the neurospheres failed to exhibit an elongated morphology; instead, these cells diminished in size and became irregularly cuboidal in shape (Fig. 2A). Forty cells each from three independent experiments were examined to measure the total area and front-to-rear ratio of cells and mitochondria. The total area of Mdivi-1 treated cells was significantly reduced when compared to the area of cells treated with DMSO (Control, 24.5 ± 11.0, vs. Mdivi-1, 25.7 ± 3.4; Fig. 2B). Mitochondrial areas were also markedly reduced (Control, 9.6 ± 5.6 vs. Mdivi-1, 2.5 ± 1.2; Fig. 2C). The ratio between front and rear compartments was 2.7 ± 1.9 in the control cells, indicating that the front/leading process was substantially larger than the rear process. Contrarily, the front-to-rear ratio of Mdivi-1-treated cells was 1 ± 1.2, suggesting that Mdivi-1-treated cells became round in shape (Fig. 2D). Interestingly, the front-to-rear ratio of mitochondria in Mdivi-1-treated cells was unaltered when compared to the DMSO group (Fig. 2E). To ensure that Mdivi-1 did not change mitochondrial polarity, we examined the location of centrosomes as a marker for the anterior region. Following Mdivi-1 treatments, a major portion of mitochondria clustered anteriorly from the nucleus, as marked by the centrosome (Fig. 2F). Collectively, these data suggest that Drp1 activity primarily affected cell shrinkage without affecting the polarized distribution of organelles.

### Migration defect in SVZ-derived aNSCs cells after Mdivi-1 treatment

**Figure 3:**
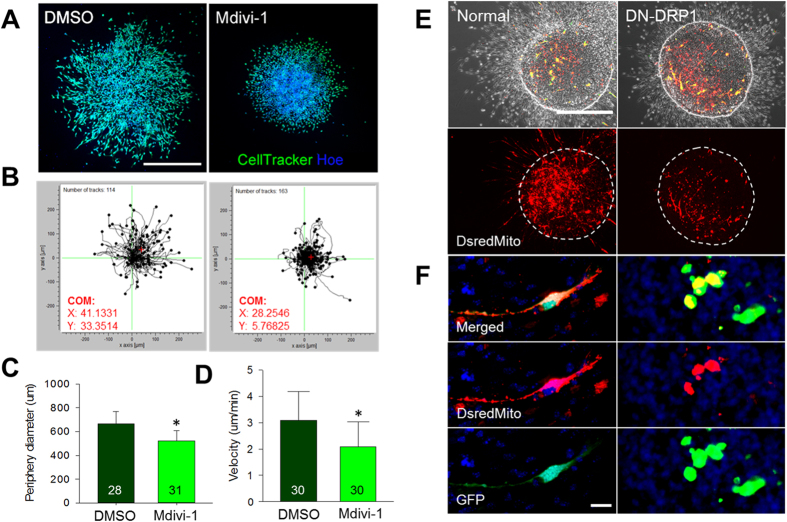
Migration of SVZ-derived cells after Mdivi-1 treatment. (**A**) Migration distance of cells from the core of neurosphere as marked by Cell Tracker (green) and nuclei (Hoechst blue). Scale bar = 50 μm. (**B**) Live imaging of individual cell behavior from the neurosphere. (**C**) The peripheral diameter shows the total distance of migration. Data are shown as the mean ± s.d.; *P < 0.05 via Student’s t-test. Numbers in bars indicate total number of analyzed neurospheres from 3 independent experiments. (**D**) Velocity to show the migration rate between two groups. Data are shown as the mean ± s.d.; *P < 0.05 via Student’s t-test. Numbers in bars indicate total number of analyzed cells from 3 independent experiments. (**E**) Cell migration following co-transfection of dsRed-mito and GFP in the normal group, and DN-DRP1 and GFP in the experimental group. Scale bar = 50 μm. (**F**) Magnified images following co-transfections show mitochondrial distribution and cell morphology. Scale bar = 10 μm.

As Mdivi-1 treatment unfavorably altered neurosphere morphology, migration distance was also significantly reduced from the core of the neurospheres (Fig. 3A). To minimize the variations, we included similar sizes (180–210 μm) of neurospheres in analyses. Live imaging of individual cell behavior confirmed the reduction, leading to an inefficient dispersion of cells from the neurospheres (Fig. 3B). The total distance of migration in the Mdivi-1 treated group was 522.87 ± 86.45 μm as compared to 664.16 ± 105.47 μm for the control (Fig. 3C). In addition, the velocity of the migrated aNSCs was also significantly reduced upon Mdivi-1 treatment when compared to the DMSO group (Fig. 3D). We further confirmed that Mdivi-1 inhibited Drp1 activity in aNSCs. Treatment of Mdivi-1 efficiently blocked thapsigargin (TG) induced mitochondria fragmentation in aNSCs (Supplementary Figure s2), we ruled out the possibility that Mdivi-1 affected cell migration independent to the suppression of Drp1 activity, as we observed that transfection of the dominant negative mutant of Drp1 (DN-DRP1) resulted in similar migration defects (Fig. 3E,F). Consistent with our previous observations, DN-DRP1-transfected cells diminished in size and failed to exhibit an elongated morphology, while mitochondria maintained polarized distribution.

### Mdivi-1 affects the fate of aNSC differentiation

Because we noticed that spontaneously differentiated DCX^+^ neuroblasts and proliferating aNSCs exhibited different mitochondrial distribution (Fig. 1B), we monitored mitochondrial localization in neurons and glial cells when removing EGF/bFGF from the media to induce differentiation. Immunostaining of the differentiated cells with class III beta-tubulin (Tuj1) for the immature neuronal cells and GFAP for the astrocytes showed striking differences in their mitochondrial localizations (Supplementary Figure 3A). The Tuj1^+^ neuronal cells exhibited mitochondria clustered near the nucleus and fragmented mitochondria along the processes. However, interconnected mitochondria were evenly distributed in the GFAP^+^ astrocytes.

**Figure 4:**
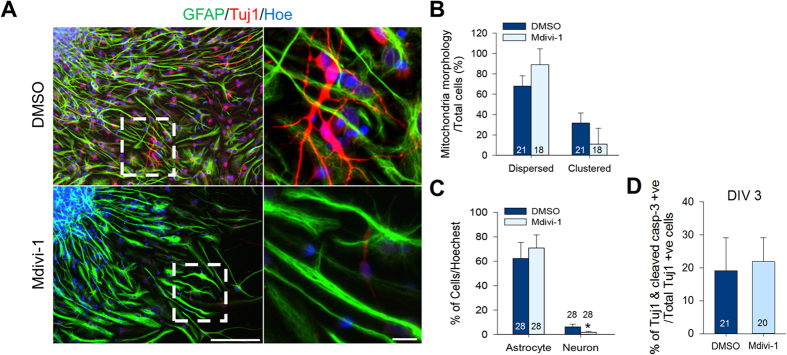
Mdivi-1 affects the fate of aNSC differentiation. (**A**) GFAP (green) and Tuj-1 (red) staining following 6 days of differentiation. Large image scale bar = 20 μm. Magnified images scale bar = 10 μm. (**B**) Quantification of mitochondrial morphology following Mdivi-1 treatment. Data are shown as mean ± s.d.; *P < 0.05 via Student’s t-test. (**C**) Quantification of astrocytes and neurons following Mdivi-1 treatment. Data are shown as mean ± s.d.; *P < 0.05 via Student’s t-test. (**D**) Quantification of Tuj1 and cleaved caspase 3 double positive cells at day 3 of differentiation. Data are shown as mean ± s.d. Numbers in bars indicate total number of analyzed cells from 3 independent experiments.

We then asked a question whether inhibiting Drp1 via Mdivi-1 affected aNSC differentiation. After treatment with Mdivi-1 on the first day of differentiation, cells were allowed to differentiate for 6 days (Fig. 4A) before categorizing them by mitochondrial morphology. For the control, approximately two-thirds of the cells exhibited dispersed mitochondria, while the rest exhibited clustered mitochondria (Fig. 4B). Conversely, Mdivi-1 treatment predominately induced dispersed mitochondria (Fig. 4B; DMSO 89 ± 15.56% vs. Mdivi-1 68 ± 10.13%). The cell population exhibiting clustered mitochondria (DMSO 31.71 ± 9.79% vs. Mdivi1 11.00 ± 15.56%) showed bipolar morphology, which was seen typically in neurons. We then quantified the effects of Mdivi-1 on aNSC differentiation and found that cells treated with Mdivi-1 normally differentiated into astrocytes (Fig. 4C; DMSO 62.23 ± 13.14% vs. Mdivi-1 73.15 ± 12.52%). Fewer cells, however, were differentiated into Tuj1^+^ neurons in the Mdivi-1 treated group in contrast to the DMSO group, which coincided with the mitochondrial morphology (Fig. 4C; DMSO 6.23 ± 2.25% vs. Mdivi-1 1.81 ± 0.72%). To further examined whether Mdivi-1 altered initial aNSCs differentiation, we allowed attached aNSCs to differentiate for 2 days and quantified the proportion of cells expressing early neuronal (DCX) and immature neuronal (Tuj1) markers. Both DCX and Tuj1^+^ cells were significantly reduced in Mdivi-1 treated group (Supplementary Figure 3B). At 3 days of differentiation, Tuj1 and cleaved caspase-3 double^+^ cells were not significantly different between Mdivi-1 and DMSO treated groups (Fig. 4D). Collectively, these data indicated that Midvi-1 prevented neuronal differentiation, but not apoptosis.

### Mdivi-1 affects the membrane potential of mitochondria and subsequent respiration

**Figure 5:**
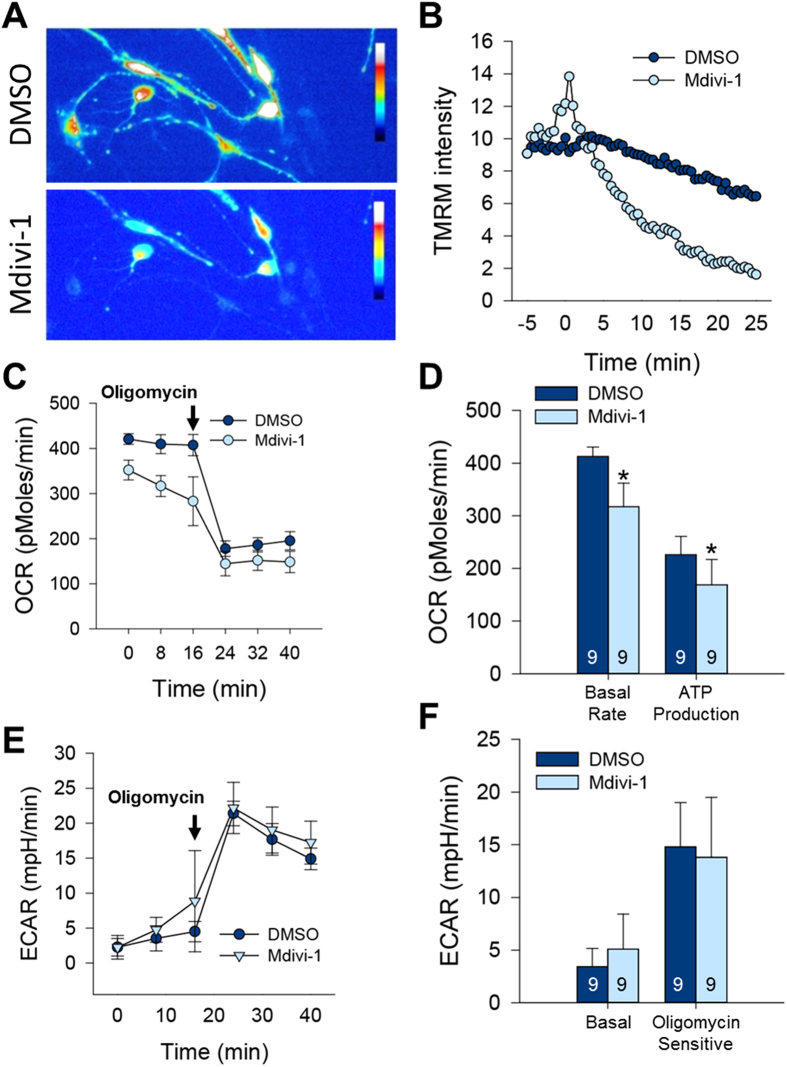
Mdivi-1 affects the respiration of mitochondria. (**A**) Representative images of TMRM after DMSO or Mdivi-1 treatment. (**B**) TMRM intensity measured in a single cell after DMSO or Mdivi-1 treatment. (**C**) Respiration measured using oxygen consumption rate (OCR). Black arrows indicate the time of oligomycin treatment. (**D**) Measurement of OCRs associated with basal rate and ATP production (**E**) Glycolysis measured by extracellular acidification rate (ECAR). (**F**) Measurement of Basal and oligomycin sensitive levels of ECAR. Numbers in bars indicate total number of examined coverslips from 4 independent experiments.

Retrograde mitochondrial transport is associated with the quality control of mitochondria, and reducing the mitochondrial membrane potential is known to activate retrograde mitochondrial transport. Furthermore, we previously observed that blocking mitochondrial fission by suppressing Drp1 promotes membrane depolarization^23^. Therefore, we next examined whether Mdivi-1 treatments perturbed mitochondrial membrane potential and ATP generation. First, we stained aNSCs with a dye for mitochondrial membrane potential, TMRM, and found a dramatic decrease in membrane potential (depolarization) after a short period (>5 min) of Mdivi-1 treatment (Fig. 5A,B). The depolarization of mitochondrial membrane is usually associated with perturbation of mitochondrial function and subsequent reduction in ATP generation. Therefore, we measured the oxygen consumption rate (OCR) of SVZ-derived aNSCs to analyze the effect of Mdivi-1 on mitochondrial respiration and ATP production. After three hours of concurrent pretreatment of Mdivi-1 or DMSO, cells were analyzed using XF24 extracellular analyzer and treated with a selective inhibitor of the electron transport chain, oligomycin (Fig. 5C). Mdivi-1 treated cells exhibited reduction in the level of basal respiration as well as ATP production in contrast to DMSO pretreated cells (Fig. 5D). We also measured the extracellular acidification rate (ECAR) as an indicator of glycolysis. In presence of oligomycin, ECAR was simultaneously increased, indicating that the aNSCs shifted mitochondrial respiration to glycolysis (Fig. 5E). However, neither basal nor oligomycin-sensitive ECAR values showed significant differences in two groups (Fig. 5F) suggesting that Mdivi-1 did not influence the cellular glycolysis.

### L-carnitine treatment restores perturbed aNSC migration and neuronal differentiation

**Figure 6:**
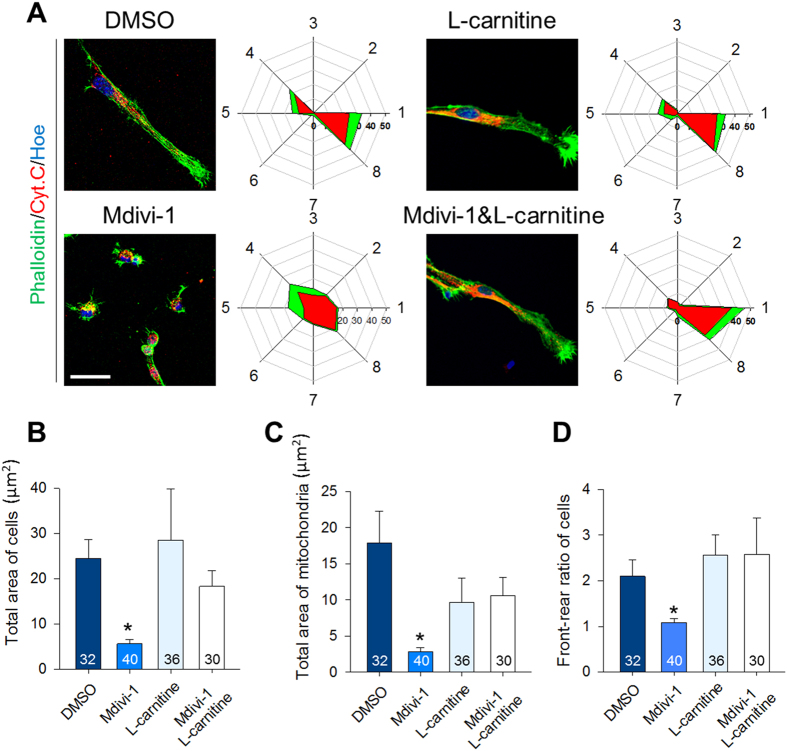
Radar plot of normalized mitochondrial distribution and cell morphology of migrating aNSCs following Mdivi-1 and/or L-carnitine treatment. (**A**) Migrating aNSCs stained with Cell Tracker phalloidin (green) and cytochrome C (red). Percentage distributions of mitochondria and cell morphology are shown in radar plotting graph. (**B**) Measurements of the total area of cells stained with phalloidin. (**C**) Measurements of the total area of mitochondria stained with cytochrome C. (**D**) Front-to-rear ratio of cells to examine polarity of cell morphology. Data are shown as mean ± s.d.; *P < 0.05 via one-way ANOVA. Scale bar = 10 μm. Numbers in bars indicate total number of analyzed cells from 3 independent experiments.

**Figure 7:**
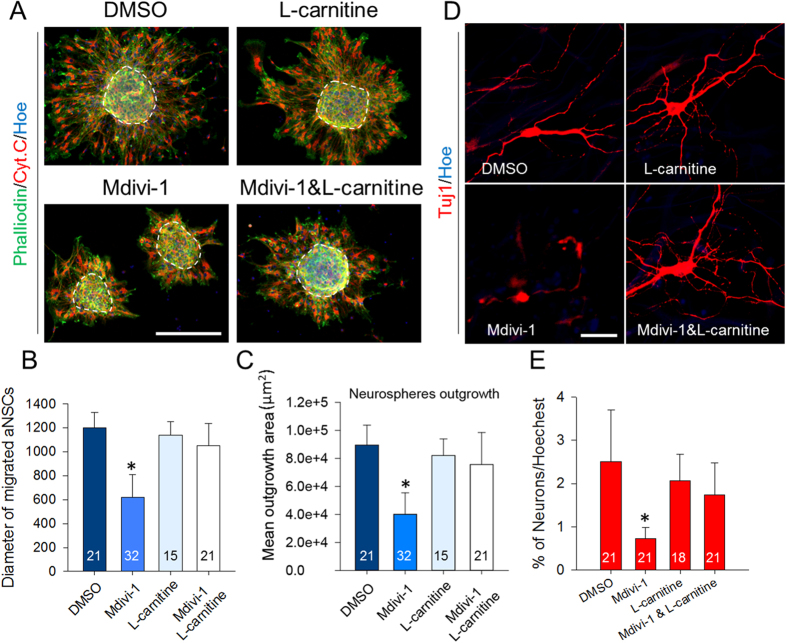
Migration and differentiation restored with L-carnitine supplementation. (**A**) Migration of attached neurospheres following DMSO, Mdivi-1, and L-carnitine treatments. Cells were immunostained with cytochrome C (Red) and phalloidin (green). Scale bar = 50 μm. (**B**) Measurements of migrated distances from the core of neurospheres. Data are shown as mean ± s.d.; *P < 0.05 via one-way ANOVA. (**C**) Measurements of neurosphere outgrowth as described in the materials and methods. Data are shown as mean ± s.d.; *P < 0.05 via one-way ANOVA. (**D**) Differentiation of neurospheres following co-treatment of L-carnitine and Mdivi-1. Magnified images scale bar = 10 μm. (**E**) Quantification of neuron percentages. Data are shown as mean ± s.d.; *P < 0.05 via one-way ANOVA Normality Test (Shapiro-Wilk). Numbers in bars indicate total number of analyzed cells from 3 independent experiments.

**Figure 8:**
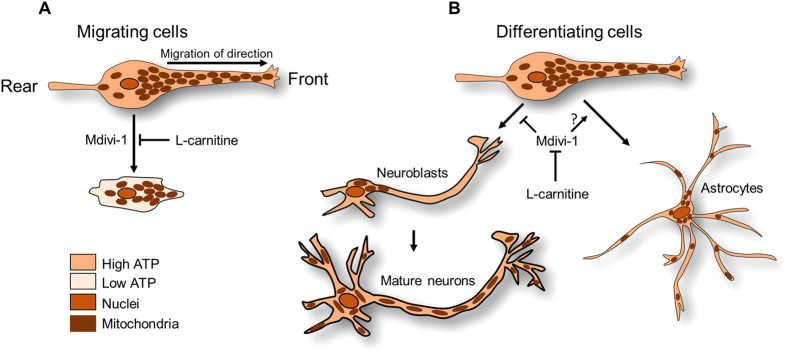
Schematic diagram of mitochondria-dependent Drp1 regulating aNSCs migration and differentiation. (**A**) Distribution of mitochondria and cell morphology before and after Mdivi-1 treatment. (**B**) Effects of Mdivi-1 on differentiating aNSCs.

L-carnitine is utilized widely to restore mitochondrial membrane potential and ATP synthesis without affecting mitochondrial morphology^23^. Thus, we asked a question whether supplementing L-carnitine with Mdivi-1 could restore the aNSC defects (Fig. 6). Consistent with our previous observation in Fig. 2, Mdivi-1 treatment promoted cell shrinkage without affecting the polarity of mitochondria. By co-treating Mdivi-1 with L-carnitine, however, the cell area (Fig. 6B), mitochondrial area (Fig. 6C), and cell polarity (Fig. 6D) were all restored to control values. Similarly, migration defects were also completely prevented by the co-treatment, as assessed by the diameters of migrated aNSCs (Fig. 7A,B). To exclude any possible variations in the different sizes of the seeded neurospheres, we measured the area covered by the outgrowth of migrating cells. The percentage of outgrowth area did not differ among DMSO, L-carnitine, or L-carnitine+ Mdivi-1 treated groups at 24 hours, but was significantly greater than the Mdivi-1 treated group (Fig. 7C). Co-treating with Mdivi-1 and L-carnitine was sufficient to restore neuronal differentiation to normalcy when compared to the DMSO group (Supplementary Figure 3 and Fig. 7D,E). Furthermore, it appeared that L-carnitine improved the arborization and hypertrophy of the differentiated neurons. Collectively, these results indicate that supplementing ATP with L-carnitine was sufficient to restore most of the defects induced by Drp1 suppression, as shown in Fig. 8.

## Discussion

NSCs in humans and mice are capable of precise migration and replacing/regenerating lost neural cells. In the adult brain, aNSCs migrate toward diseased and damaged tissues for repair/regeneration. Interneurons in the olfactory bulb are continuously regenerated by the aNSCs from the SVZ as they migrate through the rostral migratory stream (RMS) to the olfactory bulb to differentiate under the influence of their environment^27^. Conversely, during embryo development, NSC-derived progenitor cells migrate radially or tangentially to their ultimate destination^28^. The dynamic mechanisms of endogenous NSC migration and differentiation remain poorly understood. In this study, we identified for the first time that migrating aNSCs require proper Drp1 activity to maintain efficient ATP synthesis.

The cytoskeletal architecture, which is directed by kinesin and dynein motor proteins, controls the cell shape as well as mitochondrial transport and distribution^29^,^30^. As kinesin-mediated organelle transport occurs predominantly in the cell periphery, loss of kinesin activity results in perinuclear localization of mitochondria and minus-end directed dynein motor activity^31^. Pathologically, hypoxia promotes perinuclear clustering of mitochondria from inactivated Drp1, and local ROS production appears to mediate hypoxia-dependent cellular events^11^. Thus, blocking Drp1 function should promote perinuclear condensation of mitochondria in cancer cells^32^. As expected, suppressing Drp1 in the aNSCs causes mitochondria to condense in the perinuclear region, which is associated with actual cell shrinkage. However, it is important to note that the polarity of mitochondria was maintained, and measurement of front-to-rear ratio was not significantly changed by Mdivi-1 treatments. The polarized cell axis guides cell migration and the distribution of the polarized organelles. For instance, the nucleus is positioned laterally in the middle of the elongated cellular axis, while the centrosome localizes perinuclearly at the leading process. Migrating aNSCs show predominantly anterior localization of mitochondria. At the leading edge, continuous actin polymerization occurs in persistent migration and requires high ATP demand, which might explain the anterior localization of mitochondria. Therefore, it appears that the primary effect of Mdivi-1 on mitochondrial distribution is perinuclear condensation without affecting polarized localization.

There is increasing evidence that mitochondrial dynamics and localization are important in cell migration. For example, Drp1 is necessary to redistribute the mitochondria that are involved in lamellipodia formation and persistent migration in cancer cells^18^,^19^. In some cells, such as lymphocytes, uropods are formed at the rear of migrating cells, which pushes the cells in the direction of migration. The mitochondria are shown to accumulate specifically at the uropod during directed leukocyte migration in order to provide ATP^17^,^33^. Suppressing Drp1 blocked the concentration of mitochondria at the uropod, indicating that mitochondrial fission is required for the polarized distribution of mitochondria within a migrating cell. It is also known that NSC migration is different from lymphocytes, where migration is driven by modifying the cytoskeletal structures at the leading processes. In this respect, one strategy to supply ATP efficiently during cytoskeletal remodeling is to localize mitochondria to where active cytoskeletal remodeling is required for efficient migration. Consistent with our findings, we found that both chemical suppression of Drp1 using Mdivi-1 and overexpression of DN-DRP1 altered aNSC migration in association with cell shrinkage. Collectively, these data indicate that impairing Drp1 function fails to maintain mitochondrial localization, resulting in the disruption of elongated cell morphology and migration.

It is known that mitochondrial morphology and functions are dependent of cell types and functions. For example, neurons have highly elongated processes and require high amounts of cell energy as compared to other non-neuronal cells, such as astrocytes. We also found that differentiated progenitor cells from aNSC lines are associated with different mitochondrial distribution: neurons exhibit large numbers of mitochondria and rapid mobilization along the axons, whereas astrocytes have limited mitochondria. This is consistent with previous findings that showed that cell fate was determined by asymmetric mitochondrial distribution during cell proliferation^34^. Furthermore, neurons exhibit strong mitochondrial remodeling with high expression of fission-promoting enzyme Drp1^35^. Mitochondria are known to change shape during differentiation of various cell types, and Drp1 is known to play an important role in myogenic and cardiac differentiation and oocyte maturation^12^,^13^,^36^. Loss of Drp1-induced mitochondrial aggregation and irregular localization in neural cells resulted in hypoplasia and growth disturbances of the cortex and cerebellum during mouse embryonic development^37^,^38^. However, the role of mitochondria in aNSC differentiation has never been explored. Several lines of evidence suggest that mitochondria may regulate NSC differentiation. For instance, overexpression of the *dcf1* gene caused mitochondria destruction and activated the apoptosis pathway^39^. It was shown earlier that silencing *dcf1* altered NSC differentiation, leading to greater differentiation into astrocytes^40^. Interestingly, we observed that suppressing Drp1 in differentiating aNSCs impaired neuronal differentiation. Our data provide evidence that Drp1 suppression impedes proper mitochondrial localization of neural cells, which is necessary for aNSC differentiation. During initial observations, we found that a subset of cells which did not form bipolar morphology also exhibited highly concentrated mitochondria in the perinuclear region. These cells often showed multipolar morphology with DCX expression, suggesting that they were spontaneously differentiated neuroblasts. By removing growth factors to induce aNSC differentiation, we confirmed that differentiating immature neurons transiently exhibited highly condensed perinuclear morphology. Currently, it is unclear why young neuroblasts exhibit mitochondrial condensation, an event also observed when Drp1 was experimentally suppressed in aNSCs. Considering that Drp1 expression is induced in neuronal cell lines^41^, one simple explanation for this phenomena might be that Drp1 induction is transiently insufficient in immature neuroblasts and that they show Drp1-deficient phenotype. Furthermore, prolonged deficiency of Drp1 activity after Mdivi-1 treatment would eventually prevent their neuronal differentiation.

L-carnitine promotes ATP synthesis and mitochondrial hyperpolarization. Previously in our lab, we found that supplementing L-carnitine restored the altered membrane potential of mitochondria following DN-DRP1 transfection, although mitochondrial length was not changed^23^. In this study, we also found that Drp1 suppression in aNSCs de-polarized the mitochondrial membrane potential and reduced basal oxygen consumption. When we restored the cellular ATP pool via L-carnitine treatment, the impaired cell morphology, migration, and neuronal differentiation by Drp1 suppression were all restored, indicating that Drp1 suppression primarily impaired aNSC behaviors by depleting the cellular energy pool. It is known that any change in the subcellular distribution of mitochondria also influences cell function by controlling the local levels of ATP and second messengers, including ROS. For instance, protein kinase A (PKA) phosphorylates Drp1 for mitochondrial remodeling in response to growth factors or hormone signals^42^, and the linker protein, such as A-kinase anchoring protein, is known to boost mitochondrial membrane potential and mitochondrial ATP production by modulating PKA-mediated Drp1 activity^43^,^44^. Considering that cytoskeletal remodeling is an ATP-demanding process, two events mediated by Drp1 suppression - the reduction of the mitochondrial membrane potential and perinuclear retraction of mitochondria, might affect the elongated cell morphology.

Collectively, we conclude that Drp1 is important for regulating mitochondria to produce ATP for proper aNSC migration and differentiation. Our results also emphasize the central role of mitochondria in the migration and differentiation process. It would now be crucial to determine how mitochondria communicate, facilitate, and influence the molecular hierarchy that controls aNSC migration and differentiation. Our data opens a promising direction to target and manipulate mitochondria in adult neurogenesis, particularly in brain damage and diseases.

## Additional Information

**How to cite this article**: Kim, H. J. *et al*. Dynamin-related protein 1 controls the migration and neuronal differentiation of subventricular zone-derived neural progenitor cells. *Sci. Rep*. **5**, 15962; doi: 10.1038/srep15962 (2015).

## Supplementary Material

Supplementary Information
