# Supplementary material for: Dynamin-related protein 1 controls the migration and neuronal differentiation of subventricular zone-derived neural progenitor cells
Source: Sci Rep. 2015 Oct 30;5:15962. doi: 10.1038/srep15962 (PMC4626845; doi:10.1038/srep15962)
Supplement: Supplementary Information [file srep15962-s1.pdf]

Dynamin-related protein 1 controls the migration and neuronal differentiation of subventricular zone-derived neural progenitor cells

Hyun Jung Kim[1+], Mohammed R. Shaker[1+], Bongki Cho[2], Hyo Min Cho[1], Hyun Kim[1], Joo Yeon Kim[1*], Woong Sun[1*]

[1] Department of Anatomy, Brain Korea 21 Plus Program, Korea University College of Medicine, Seoul, 136-705, Republic of Korea

[2] Department of Brain Science, Daegu Gyeongbuk Institute of Science and Technology, Daegu, Republic of Korea

+ These authors contributed equally to this work.

***Co-corresponding author**:

Woong Sun, Ph.D.

Department of Anatomy, Korea University College of Medicine,

Anam-dong, Sungbuk-gu, Seoul, Korea

E-mail: woongsun@korea.ac.kr

Tel: +82-2-2286-1404; Fax: +82-2-929-5696

Joo Yeon Kim, Ph.D.

Department of Anatomy, Korea University College of Medicine,

Anam-dong, Sungbuk-gu, Seoul, Korea

E-mail: eleneu@korea.ac.kr

Tel: +82-2-2286-1393; Fax: +82-2-929-5696

Supplemental Information

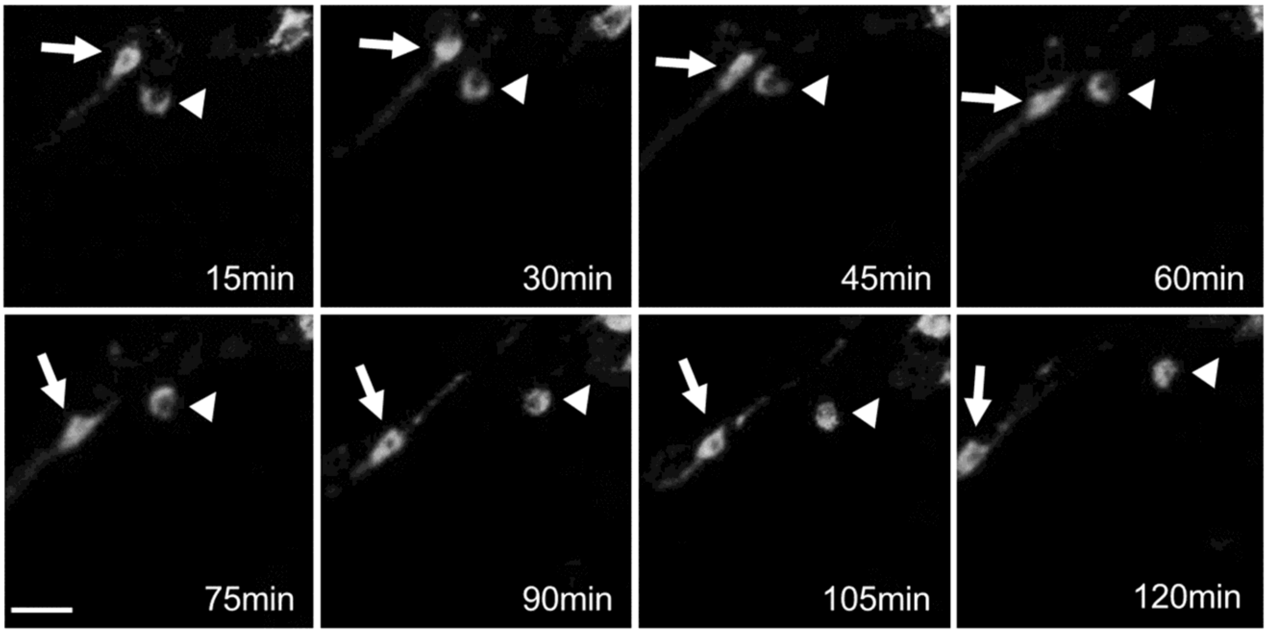

Supplementary Figure S1. Live imaging of NSC migration. NSCs electroporated with DsRed-mito and allowed to generate neurospheres. Attached neurospheres were recorded in 15 min intervals for 2 hours. Scale bar = 10 μm.

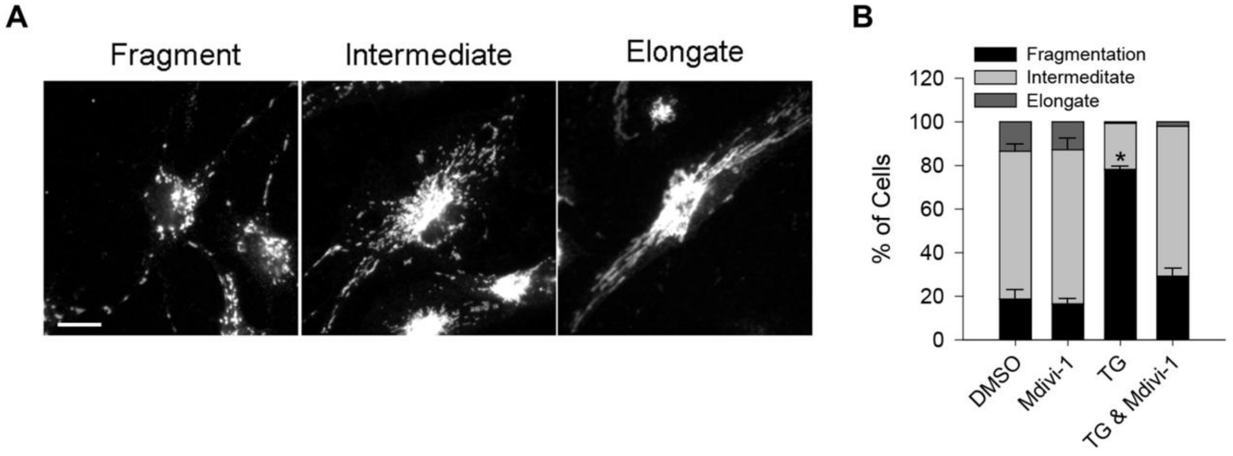

Supplementary Figure S2. Mitochondria induced fragmentation. **(A)** Representative images of mitochondria types. Scale bar = 10 μm. **(B)** Quantification of Mitochondria upon DMSO, Mdivi-1, thapsigargin (TG) and Mdivi-1 and TG treatments. Data are shown as mean±s.d. with n = 3; *P > 0.05 via one-way ANOVA.

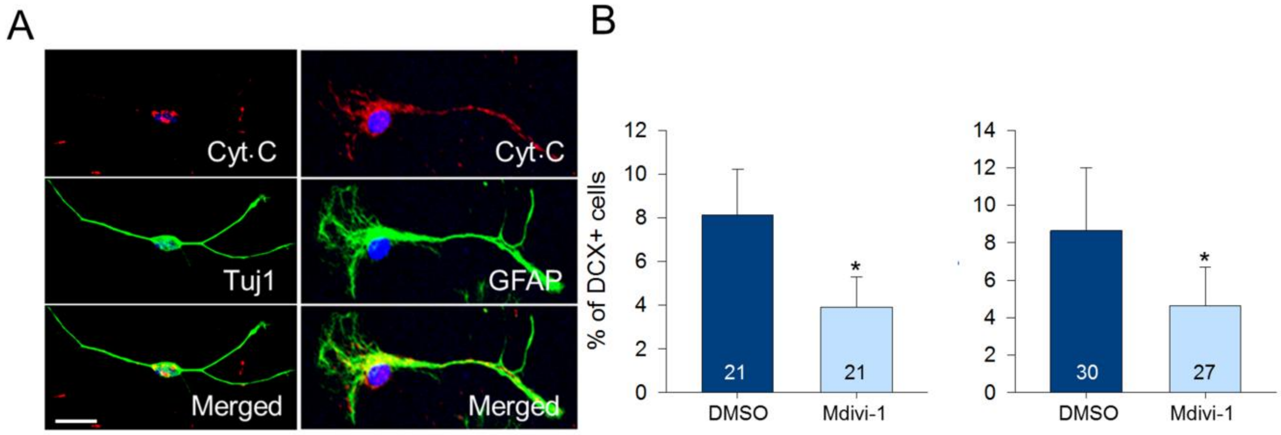

Supplementary Figure S3. Immunostaining of NSCs differentiation. **(A)** Mitochondria were marked by cytochrome C (red) co-labeled with GFAP (green) or Tuj1 (green) following 6 days of differentiation without Mdivi-1 treatment. **(B)** Quantification of DCX and Tuj-1 +ve cells following 2 days of differentiation. Data are shown as mean±s.d.; *P < 0.05 via Student's t-test. Scale bar = 10 μm. Numbers in bars indicate total number of analyzed neurospheres from 3 independent experiments.

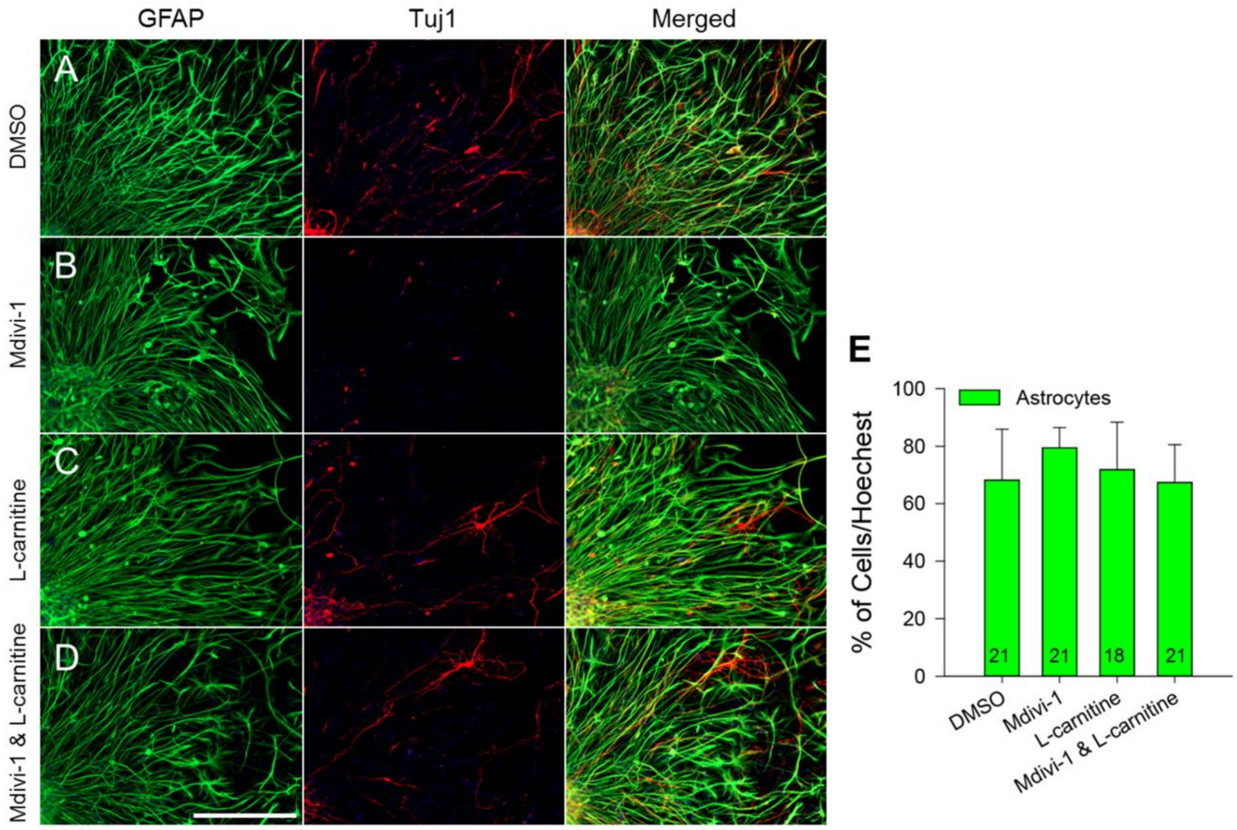

Supplementary Figure S4. Differentiation of astrocytes following by L-carnitine treatment. Differentiation of neurospheres following: **(A)** DMSO treatment, **(B)** Mdivi-1 treatment, **(C)** L-carnitine treatment, and **(D)** Mdivi-1 & L-carnitine treatments. Scale bar = 30 μm. **(E)** Quantifications of differentiated astrocytes. Data are shown as mean±s.d.; *P > 0.05 via one-way ANOVA. Numbers in bars indicate total number of analyzed neurospheres from 3 independent experiments.
